# The Latest Advances in the Diagnosis and Treatment of Dementia

**DOI:** 10.7759/cureus.50522

**Published:** 2023-12-14

**Authors:** Rehab Hafiz, Lama Alajlani, Albatool Ali, Ghadah A Algarni, Hassan Aljurfi, Omar Abdullah M Alammar, Maria Y Ashqan, Alanoud Alkhashan

**Affiliations:** 1 Family Medicine, Al Takassusi Primary Healthcare Center, Makkah, SAU; 2 College of Medicine, King Saud Bin Abdulaziz University for Health Sciences, Jeddah, SAU; 3 College of Medicine, Fakeeh College for Medical Sciences, Jeddah, SAU; 4 Family Medicine, Alfath Care Center, Madinah Health Cluster, Ministry of Health, Madinah, SAU; 5 Internal Medicine, King Fahad Hospital, Al Hofuf, SAU

**Keywords:** personalized medicine, major neurocognitive disorder, alzheimer’s disease, acetyl-cholinesterase inhibitors, lewy body dementia

## Abstract

Dementia is a debilitating neurological condition that is characterized by persistent cognitive decline. It is a global health challenge, with a rapidly increasing prevalence due to an increasing aging population. Although definitive diagnosis of various conditions of dementia is only possible by autopsy, clinical diagnosis can be performed by a specialist. The diagnostic process has evolved with recent breakthroughs in diagnostic tools, such as advanced imaging techniques and biomarkers. These tools facilitate early and accurate identification of the condition. Early diagnosis is vital, as it enables timely interventions to improve the quality of life for affected individuals. Treatment strategies for dementia encompass both pharmacological and non-pharmacological approaches. Non-pharmacological treatments include cognitive training and lifestyle modifications. Among pharmacological treatments, acetyl-cholinesterase inhibitors including donepezil, rivastigmine, and galantamine can be used in various doses based on the severity of the disease. Apart from these, N-methyl-D-aspartate receptor antagonists such as memantine can also be used. Furthermore, personalized treatments have also gained significant attention in dementia treatment. Interdisciplinary care, involving healthcare professionals, social workers, and support networks, is crucial for comprehensive and holistic dementia management.

## Introduction and background

According to the World Health Organization (WHO), dementia is classified as a syndrome rather than a disease [1]. It is characterized by a progressive decline in the cognitive function of an individual across multiple cognitive domains, consequently impairing functional abilities. The term dementia has been replaced by major neurocognitive disorder (MND) in the Diagnostic and Statistical Manual of Mental Disorders-5 (DSM-5) [2]. However, due to the higher recognition of the term dementia, we will use it in this review. 

The cognitive decline seen in dementia is usually associated with the prior level of cognition of the patient. Furthermore, the decline is often persistent and is not associated with an isolated episode of delirium. The most frequent symptoms of dementia include progressive impairment in thinking, language, memory, learning, and judgment, difficulty in concentrating, being confused about time and place, and mood changes [3,4]. According to the DSM-5, there are 13 etiologies that can contribute to dementia. However, Alzheimer's disease (AD) is the underlying etiology in approximately 70% of all cases [5]. Other causes of dementia include Lewy body disease, traumatic brain injury, vascular disease, and frontotemporal lobar degeneration [6].

Several risk factors have been identified for dementia, such as being over the age of 65 and having hypertension, diabetes, smoking, and depression [4,5]. These factors make it more likely for older adults to develop dementia. With the growing population of individuals over 65, the number of dementia patients is expected to increase in the future [5]. Additionally, the prevalence of dementia has been linked to socioeconomic status and culture, with a higher occurrence seen in countries with lower and middle incomes [3,6].

## Review

Prevalence of dementia

According to WHO, almost 55 million people suffer from dementia across the globe [7]. The prevalence of dementia has been associated with socioeconomic level and culture [8]. The majority of dementia patients are from low-and middle-income countries. Furthermore, almost 10 million new cases are being added every year [7]. Among various risk factors, age above 65 years, hypertension, diabetes, smoking, and depression are the main contributors. Older adults are particular victims of this condition, as it is the most prevalent condition among neurological diagnoses in this age group [4,9]. As the number of individuals aged above 65 is increasing, the number of dementia patients is expected to increase to 131.5 million by the year 2050 [9]. A meta-analysis by Cao et al. reported that the prevalence of dementia is 244 times higher in individuals aged over 100 years compared to the 50-59 years age group. Furthermore, they revealed that the number of dementia patients doubles every five years [10]. The prevalence of dementia also varies according to the region. For example, a systemic review showed that there was a greater incidence in Latin America (8.5%) and a noticeably lower incidence in the four sub-Saharan African regions (2-4%) [11]. Similarly, a cross-sectional study from Saudi Arabia that included 1613 participants showed that 16.6% of older individuals were suffering from dementia [6].

Pathophysiology of dementia

The pathophysiology of dementia varies according to the sub-type of dementia. For example, gross examination of the brain of patients with AD indicates lower brain weight, which can be 100-200 g below average or higher depending on the severity of the disease. The temporal, frontal, and parietal regions show cortical atrophy. However, the thalamus, brainstem, cerebellar hemispheres, and basal ganglia typically show normal size and weight. Furthermore, senile plaques and neurofibrillary tangles are visible under a microscope [12]. Extracellular plaques consist of beta-amyloid, whereas intracellular neurofibrillary tangles are composed of hyperphosphorylated tau cytoskeletal filaments. The nucleus basalis, cortex, amygdala, and hippocampus are typically where these changes occur. The degree of clinical illness and cognitive deterioration is inversely correlated with the number of tangles. The damage that beta-amyloid causes to brain cells includes intracellular calcium accumulation, oxygen radical formation, nitric oxide synthesis, and inflammatory processes. The basal forebrain has injured cholinergic neurons, resulting in a diminished level of cholinergic neurotransmission. Further findings include the degeneration of the locus caeruleus and raphe nuclei, which leads to deficits in the neurotransmitters glutamate, noradrenaline (norepinephrine), serotonin, and corticotropin-releasing factor [12-14].

Similarly, different types of changes are seen in other types of dementia as well. Lewy body dementia (LBD) is characterized by the accumulation of aggregates of alpha-synuclein protein, called Lewy bodies [15]. HIV-associated dementia can cause neurodegeneration of the brain due to toxic inflammation by HIV [16]. Dementia due to alcohol consumption can be caused by cytotoxic processes [17]. 

Diagnosis of dementia

The definitive diagnosis of the various types of dementia is only possible by the autopsy of the patient. However, a clinical diagnosis of dementia can be made by a specialist in a primary care setting by clinical examination of the patient. Furthermore, neuroimaging, biomarkers, and digital tools can also be used by the physician. Early diagnosis of dementia is crucial for timely intervention and management of the disease. In recent decades, the diagnostic tools and techniques for dementia have improved significantly.

*Neuroimaging* 

Although diagnostic evaluations are primarily performed based on clinical criteria, the role of neuroimaging has also expanded significantly [18]. Advanced neuroimaging techniques, such as diffusion tensor imaging (DTI), functional MRI (fMRI), positron emission tomography (PET), and single-photon emission computed tomography (SPECT), have been shown to provide valuable information in the diagnosis of dementia. DTI can detect changes in the structural connectivity of white matter tracts, which can indicate the presence of dementia [19]. The use of fMRI can detect changes in brain activity patterns, which can indicate functional alterations in the brain associated with dementia [20]. PET and SPECT can detect changes in brain metabolism and blood flow, improving the accuracy of the diagnosis of dementia [21] Some studies have investigated the use of novel PET tracers that target specific pathological features of dementia, such as beta-amyloid and tau proteins [22]. Machine learning algorithms that analyze neuroimaging data have also been developed to aid in the diagnosis of dementia [23].

*Biomarkers* 

Recent studies have investigated the use of blood-based biomarkers, such as neurofilament light chain (NfL) and plasma phosphorylated tau (p-tau), for the detection of neurodegeneration in the brain. Elevated levels of these biomarkers indicate the presence of dementia, even before symptoms appear [24]. The use of blood biomarkers is potentially helpful in diagnosing the pathological features of AD. In addition to blood-based biomarkers, CSF biomarkers, such as amyloid beta, tau, and phosphorylated tau, have been shown to improve the accuracy of the diagnosis of AD [25]. Retinal biomarkers, such as retinal nerve fiber layer thickness and macular volume, have also been investigated as potential biomarkers for the early diagnosis of dementia [26]. 

Digital Tools

The modern healthcare industry has embraced technological advancements to improve the provision of care. Mobile-based applications technology is one such innovative and trending invention that supports increased patient care management and quick diagnostics [27]. Digital tools, such as artificial intelligence (AI) machine learning algorithms, smartphone apps, and wearable devices, have gained traction in the diagnosis of dementia. These tools can detect subtle changes in cognitive function and behavior, which can indicate the presence of dementia. AI algorithms can accurately predict the onset of AD even before symptoms appear, by analyzing neuroimaging data [28]. Smartphone apps such as Sea Hero Quest (Glitchers, Edinburgh, Scotland) have been developed to assess spatial navigation skills, which are often impaired in the early stages of dementia [29]. Wearable devices such as smartwatches and fitness trackers have also been used to monitor changes in physical activity and sleep patterns, which can be potential early indicators of dementia. BrainCheck Inc. (Austin, Texas, United States) has developed five distinct games designed based on gold-standard neurocognitive testing. Based on the outcomes of each game, the app shows a graph that indicates the degree of cognitive function relevant to executive function, cognitive processing, immediate memory, visual attention, and delayed recall skills of the user [30]. Early diagnosis of dementia is critical for timely intervention and management of the disease [31]. 

Management of dementia

Due to the neuronal cell loss in dementia, there is no curative treatment available so far. Therefore, symptomatic management remains the mainstay of treatment: treatment of behavioral disturbances, environmental manipulations that support function, and counseling about safety concerns [32]. The main aim of dementia management is to delay cognitive decline and relieve the patient from cognitive suffering. Both non-pharmacologic and pharmacologic approaches are employed, either alone or in combination. The current treatments for dementia vary based on the types of dementia. Two main types of medication have been approved for the treatment of dementia including cholinesterase inhibitors and memantine. AD-related dementia can be treated with acetylcholinesterase inhibitors (AChEIs) including donepezil, rivastigmine, and galantamine in various doses based on the severity of the disease. Apart from these, N-methyl-D-aspartate (NMDA) receptor antagonists such as memantine can also be used. Other types of dementia such as vascular dementia, Parkinson's disease dementia, Down syndrome dementia, LBD, and frontotemporal dementia can also use these pharmacological options. 

AChEIs

It has been shown that AChEIs such as galantamine, rivastigmine, and donepezil increase cholinergic transmission by halting cholinesterase at the synaptic cleft while providing modest symptomatic relief in certain patients suffering from dementia. It is the mainstay drug used in AD patients as they have lower cerebral content of choline acetyltransferase, resulting in diminished acetylcholine production and poor cortical cholinergic function. Donepezil has been found to significantly improve cognition, clinical assessment, and functional outcomes in the higher-dose group [33-36]. AChEIs are presently the gold standard for treating LBD-related cognitive and psychiatric symptoms. Rivastigmine is the only one of the three that is FDA-approved for treating LBD [37-40]. The remaining two are used off-label. There is no compelling evidence that one of the three is more effective than the others in treating LBD [41-43]. There is limited data on the use of AChEIs in treating frontotemporal dementia, and the available ones do not support the use of AChEIs in frontotemporal dementia [44]. The treatment of dementia in Parkinson's disease centers on the use of AChEIs. Most, but not all, trials with AChEIs in Parkinson's disease dementia found a slight to moderate benefit, albeit at the expense of an increased risk of adverse effects such as exacerbated tremors and nausea [45,46].

Memantine

Memantine is an NMDA receptor antagonist. In contrast to cholinergic agents, memantine acts in a neuroprotective manner. Cortical and hippocampal neurons' main excitatory amino acid neurotransmitter is glutamate. Moreover, the NMDA receptor, which is involved in memory and learning, is one of the receptors that glutamate activates. It is currently used in treating AD jointly with AChEIs, especially in advanced AD. Agents that block pathologic stimulation of NMDA receptors may also prevent further harm in patients with vascular dementia (VaD), as excessive NMDA stimulation can be generated by ischemia and result in excitotoxicity. The residual neurons' physiological function might also be recovered, improving symptoms [47]. Many clinicians are turning to memantine because of the lack of other established treatments for VaD. Memantine is frequently used in conjunction with AChEIs in people who can afford it. According to the 2020 guidelines for treating LBD in general, AChEIs have the most evidence for usage in cognitive impairment, whereas memantine has mixed evidence [39]. There was no statistically significant difference in the usage of memantine in FTD [44]. Memantine was well-tolerated in some trials in patients with PDD. However, hallucinations and worsening neuropsychiatric symptoms have been recorded using memantine, indicating that it should be used cautiously in PDD [48]. 

Antioxidants

Vitamin E (alpha-tocopherol) and selegiline (a monoamine oxidase inhibitor) have been studied for their potential in treating AD. Research has shown that vitamin E, when administered at a specific dose, may provide a slight reduction in functional decline in patients with mild to moderate AD. However, its impact on cognitive function is not considered significant [49,50]. On the other hand, medications targeting serotonergic pathways, such as selective serotonin reuptake inhibitors (SSRIs) and trazodone, as well as atypical antipsychotic treatments, have been found to effectively manage specific behavioral symptoms associated with AD. However, it is important to note that these medications do not improve overall cognitive function. In the case of frontotemporal dementia, SSRIs like sertraline and fluvoxamine have shown positive effects on impulsivity, eating disorders, and anxiety based on case reports and short observational studies [51].

Most Recent Drugs: Lecanemab and Aducanumab

Recent advancements in the field of dementia treatment emphasize the significance of focusing on the underlying pathological mechanisms of the disease, like amyloid-beta plaques. By directly addressing these crucial pathophysiological characteristics of AD, both lecanemab and aducanumab have the potential to provide therapeutic benefits by slowing cognitive decline and enhancing measures of cognition and function in affected individuals.

The FDA has recently approved lecanemab, an anti-amyloid monoclonal antibody, for treating mild cognitive impairment (MCI) and mild dementia caused by AD [52]. This approval represents a significant breakthrough in dementia treatment. Lecanemab specifically targets the underlying causes of AD by binding to amyloid-beta plaques, which are one of the main pathological characteristics of the disease [53,54].

A notable study conducted by van Dyck et al. demonstrated the effectiveness of lecanemab in reducing amyloid markers in individuals with early-stage AD. The study showed that treatment with lecanemab resulted in a slower deterioration in measures of cognition and function over 18 months compared to a placebo [55]. However, it is important to note that adverse events were a major concern following treatment with lecanemab, highlighting the need for careful monitoring and management of potential side effects [55].

Another study by McDade et al. further supported the efficacy of lecanemab in reducing brain amyloid and slowing cognitive decline in patients with AD. The treatment regimen involved administering lecanemab at a dosage of 10 mg/kg biweekly [56]. These findings provide additional evidence for the potential benefits of lecanemab in managing the progression of AD.

Similarly, the efficacy of another anti-amyloid-beta monoclonal antibody, aducanumab, has been extensively documented in the literature. A systematic review conducted by Rahman et al. reported that the use of aducanumab led to a reduction of amyloid-beta plaques and a significant decrease in cognitive decline among AD patients [54]. The approval of aducanumab by the FDA in June 2021 further supports its effectiveness as a treatment option for AD.

Non-Pharmacological Treatments 

Although there is no single dietary intervention that has definitively shown to effectively prevent cognitive degeneration and dementia, it is important to consider various factors that can contribute to maintaining brain health and reducing the risk of AD. Along with maintaining a healthy and balanced diet, regular physical activity, sufficient sleep, and stress management are all important lifestyle factors that have been associated with a decreased risk of cognitive decline [57,58].

Mediterranean Diet (MedDiet) and Dietary Approaches to Stop Hypertension (DASH):

In prospective observational studies and trials, the MedDiet has been consistently seen to provide numerous benefits in preventing various non-communicable diseases. This includes cognitive decline and dementia, which are major concerns in aging populations. The MedDiet is characterized by a high consumption of plant-based foods such as fruits, vegetables, whole grains, legumes, nuts, and olive oil. It also involves moderate intake of fish and poultry, and limited consumption of red meat and processed foods. The diet is rich in nutrients such as antioxidants, omega-3 fatty acids, fiber, and vitamins, which have been associated with cognitive health [59,60].

Several studies have shown that individuals who closely follow the DASH and MedDiet, while also consuming more whole grains, nuts, and legumes, tend to achieve higher scores on the Mini-Mental State Examination (MMSE), a widely used cognitive assessment tool [59-62]. The MMSE evaluates various cognitive domains such as orientation, memory, attention, and language, with higher scores indicating better cognitive function. These findings suggest a strong association between a healthier dietary pattern and improved cognitive function. Furthermore, a systematic review conducted by Lourida et al. provides additional support for the positive impact of the MedDiet on cognitive health. The review analyzed multiple studies and concluded that adherence to the MedDiet is linked to a slower rate of cognitive decline and a reduced risk of AD [63].

The potential mechanisms underlying the beneficial effects of the MedDiet on cognitive health are multifactorial. The high intake of fruits, vegetables, and whole grains provides a rich source of antioxidants. These antioxidants help combat oxidative stress and inflammation, which are processes believed to contribute to cognitive decline. The MedDiet is also rich in omega-3 fatty acids, mainly from fish consumption, which have been associated with improved cognitive function and a lower risk of dementia. Additionally, the MedDiet promotes cardiovascular health by reducing the risk of hypertension, diabetes, and obesity. These conditions are known to increase the risk of cognitive impairment [61-63].

Ketogenic Diet

The ketogenic diet has been proposed as a potentially neuroprotective approach against cognitive decline. This diet is characterized by a low carbohydrate composition, moderate protein consumption, and high-fat consumption. In recent years, it has gained attention for its potential in preventing cognitive decline associated with aging. However, it is important to note that there is currently insufficient clinical trial data to draw definitive conclusions about the effectiveness of the ketogenic diet in preventing and treating cognitive decline and AD [64,65]. A systematic review conducted by Devranis et al. included seven studies on the ketogenic diet and reported that it may have the potential to reduce cognitive decline [66]. However, it is crucial to consider the limitations of the existing research. The number of studies available is relatively small, and the quality and design of the studies vary.

The ketogenic diet is believed to have neuroprotective effects due to several mechanisms. Firstly, the diet's low carbohydrate composition induces ketosis, a state where the body produces ketone bodies as an alternative fuel source for the brain. Ketone bodies, like beta-hydroxybutyrate, are believed to possess neuroprotective properties and offer an alternative energy source for brain cells, potentially enhancing their function [65].

Additionally, the ketogenic diet has been shown to reduce inflammation and oxidative stress, which are believed to play a role in the development and progression of neurodegenerative diseases. By limiting the intake of carbohydrates and promoting the consumption of healthy fats, the diet may help reduce the production of reactive oxygen species, which can damage brain cells and contribute to cognitive decline [65].

Furthermore, the ketogenic diet has been found to modulate various signaling pathways and gene expressions that are involved in neuronal health and function. For example, it has been shown to activate pathways that promote the synthesis of neurotrophic factors, such as brain-derived neurotrophic factor (BDNF), which supports the growth and survival of neurons [66]. The diet may also enhance mitochondrial function and increase the production of adenosine triphosphate (ATP), the primary energy currency of cells, which is essential for proper brain function [64].

Physical Activity

According to recent research, increasing physical activity levels has been found to have a preventive effect on approximately 3% of all dementia cases [67,68]. Additionally, engaging in physical activity and exercise has been shown to improve overall cognitive function in individuals with dementia [69]. This positive impact is likely attributed to multiple underlying processes. Firstly, physical activity and exercise help in managing cardiovascular risk factors that are associated with impaired cognitive performance. By promoting cardiovascular health, physical activity contributes to maintaining optimal brain function [70]. Furthermore, animal studies have demonstrated that physical activity and exercise can stimulate neurogenesis, the generation of new neurons, and synaptic plasticity, the ability of neurons to form connections with one another. These processes are crucial for maintaining brain health and cognitive function [70,71].

Sleep Patterns

Sleep disturbance is not only a symptom but also a risk factor for neurocognitive conditions, such as dementia [72]. Adequate sleep is now recognized as crucial for memory consolidation and the removal of excess beta-amyloid and hyperphosphorylated tau, which are characteristic biomarkers of AD. Sleep difficulties often precede the onset of AD pathology and cognitive decline [73]. Non-pharmacological sleep therapies have shown promise in improving sleep quality and may positively impact cognitive function in individuals with dementia. Multidomain approaches that address various aspects of sleep hygiene, including the sleep environment, bedtime routines, and relaxation techniques, have shown particular effectiveness [73,74]. However, it is important to note that the existing research on non-pharmacological sleep therapies for dementia is heterogeneous and limited.

Challenges of caring for individuals with dementia

There are multiple challenges in caring for patients with dementia. Behavioral changes such as agitation, aggression, delusions, and hallucinations make it difficult to provide care for patients with dementia. Agitation and aggression are caused by neuroleptic overdose, internal medical conditions, or pain. Cognitive deficit occurring in dementia patients makes it hard for them to express pain and in turn, it manifests as a state of agitation [75]. Sleep disturbance with increased nighttime wakefulness and reduction in the total amount of sleep time is associated with cognitive decline [76]. Another issue is safety, with later stages of dementia patients wandering and becoming lost is one problem that faces caregivers. [77] One more thing to consider is eating problems, which are common among demented patients and require nutritional support [78].

It is important to face the different challenges facing dementia patients' caregivers with a more holistic point of view taken towards dementia with an interdisciplinary team approach. Interventions should be individualized according to the needs of the patients. The effectiveness of psychosocial interventions tailored to the patient's needs for the management of neuropsychiatric symptoms has been demonstrated [79]. An essential aspect of effective management involves rehabilitation, which entails a collaborative effort with healthcare professionals to adopt a patient-centered approach that also incorporates the involvement of caregivers [80]. According to the WHO, rehabilitation is important to meet the needs of the affected people. Older adults and their caregivers have the potential to live an active and social life with interdisciplinary rehabilitation [80]. 

There is a plethora of challenges that caregivers of patients with dementia face. One example is that caregivers constantly need to be alert and involved in the medical treatment of dementia patients due to their cognitive impairment [81]. Care recipients may not value the assistance provided by their caregivers and sometimes even refrain from their medical treatment [82]. Several studies reported that patients sometimes believed that their given medications were poisonous and that they became doubtful and paranoid when given their treatment [82,83]. Another challenge is the behavioral changes of elderly patients with dementia that sometimes lead them to refuse care. Some patients lose insight regarding their condition and need for food or medications. As a result, it has been reported in a study that nutritional care may be complicated for patients with dementia as they can accumulate food or become hostile at mealtime [84]. Also, caregivers for dementia patients deal with complex treatment regimens. A study reported that care recipients often had around nine comorbidities and received more than 10 medications [85]. Lastly, data shows that caregivers did not receive enough information and teaching regarding their medical and nursing tasks. Several studies also showed that the lack of education and training for caregivers was attributed to the limited time available with the healthcare providers [82,86]. 

The treatment approach to people with dementia is complicated as they present with symptoms in multiple domains. These include impaired cognition, neuropsychiatric symptoms, daily activities, and often other medical and comorbidities. Interventions to treat dementia patients must consider their cognitive, physical, emotional, and psychosocial needs [67]. Thus, elderly people with dementia require rehabilitation involving several healthcare professionals to improve their independence to perform their daily activities. One study demonstrated that patients who received interdisciplinary care had stable and slightly improved cognitive status and performance of activities of daily living [87].

## Conclusions

Dementia is a significant contributor to morbidity in elderly individuals. As the number of individuals aged above 65 years is increasing, there is also an increase in dementia cases, and it has emerged as a significant concern for healthcare systems and communities across the globe. However, the latest advances in the diagnosis and treatment of dementia have ushered in a new era of hope and progress in the battle against this debilitating condition. New diagnostic tools have helped early diagnoses of dementia. Currently, research is ongoing both on the pharmacological and non-pharmacological treatment of dementia. In the journey to combat dementia, knowledge is our greatest weapon. As we move forward, collaboration between researchers, healthcare providers, and caregivers is key to managing dementia.
